# On the Curative Effects of Carriage and Travelling Exercise

**Published:** 1829-04-01

**Authors:** 


### ON THE CURATIVE EFFECTS OF CARRIAGE AND TRAVELLING EXERCISE. By M. CORIOLIS; WITH OBSERVATIONS, by DR. JOHNSON.

"Longum iter est per præcepta---breve et efficax per exempla." *Seneca.*

The author of this paper, which is published in a late number of Magendie's Journal, is an engineer, but evidently a man of science, as well as sense. Being also an invalid, he took the advice of a celebrated Parisian physician, who recommended him carriage-exercise, for the cure of a nervous complaint with which he was afflicted. M. Coriolis set about making a number of experiments on the different kinds of exercise, and on the effects of these on the human body, its functions, and its disorders. There is a general impression on all minds that exercise is conducive to health—and the impression is founded in truth; but there is considerable diversity in the modes and effects of exercise—and that which would cure one disease might aggravate another.

The author justly observes, that exercise produces two very distinct effects; —that which results from the expenditure of power—and that which results from the movements of different parts of the body. These effects can be produced either separately or simultaneously. They are both effected in walking, and in almost every species of active exercise. The expenditure of force can be effected singly, as, for instance, in the support of a burthen, where no *motion* is given to the muscles or other parts of the body. The same expenditure is produced by mental labour, without exercise of the body. This last kind of expenditure generally leads to exhaustion and debility. It is a common cause, in civilized life, of various diseases, and requires counteracting remedial agencies. To give movement to the various parts of the body, *without expenditure of power*, the carriage is the best vehicle, though our author includes horsemanship, which, however, requires the action of many muscles. We agree with M. Coriolis, that passive exercise is productive of excellent effects in various chronic diseases. He thinks that these good effects are not to be solely ascribed to the change of air, and the amusement of travelling; but that much is attributable to the passive or oscillatory motion communicated to the different structures of the body. Such is the conviction produced by experiments made in his own person. He remarks, that travelling in some kinds of voitures, causes illness—at least sickness, rather than restoration of strength—while conveyance by a different kind of machine has an opposite effect. So, again, the effects of a sea voyage, where there is little motion of muscles, though plenty of fresh air, are very different from travelling in a convenient carriage, where there is more of the succussive or passive motion communicated to the body. We daily observe people living in the purest air of the country, with abundance of mental amusement, whose health is bettered, and whose strength is recruited, by travelling from home, even in carriages with the windows closed. This, he observes, proves the utility of passive motion. M. Coriolis makes many judicious remarks on the effects of carriage-exercise, as modified by the construction of the vehicle. The swinging of the easy Berlin is far less salutary than the quick and rapid vibrations of common stages, where the springs are short, and the velocity of movement considerable. The author, after these considerations, comes to the conclusion, or rather to the proposition, that machines should be constructed, and worked by steam, at a small expense, in which invalids might have all the vibratory movements and beneficial effects of stage-coach travelling, without going out of the environs of Paris. Here we are forced to disagree with M. Coriolis. We are willing to concede to his plan all the good effects, and they are many, resulting from the passive or vibratory motion of travelling vehicles; but observation somewhat more extensive than that of M. Coriolis, convinces us that these passive movements, imparted by the most ingenious steam-engine, would prove a very poor substitute for that combination of salutary effects, moral and physical, which results from travelling. The following extracts from a work recently published by the Editor of this Journal, and which, for obvious reasons, could not be *reviewed*, will probably be pardoned, as it is the only notice the work has ever received in the Medico-Chirurgical Review.

Travelling.

"*Physical Effects.* The first beneficial influence of travelling is perceptible in the state of our corporeal feelings. If they were previously in a state of morbid acuteness, as they generally are in ill health, they are rendered less sensible. The eye, which was before annoyed by a strong light, soon becomes capable of bearing it without inconvenience; and so of hearing, and the other senses. In short, morbid sensibility of the nervous system generally is obtunded or reduced. This is brought about by more regular and free exposure to all atmospheric impressions and changes than before, and that under a condition of body, from exercise, which renders these impressions quite harmless. Of this we see the most striking examples in those who travel among the Alps. Delicate females and sensitive invalids, who, at home, were highly susceptible of every change of temperature and other states of the atmosphere, will undergo extreme vicissitudes among the mountains, without the smallest inconvenience. I will offer an example or two in illustration. In the month of August, 1823, the heat was excessive at Geneva and all the way along the defiles of the mountains, till we got to Chamouni, where we were, at once, among ice and snow, with a fall of 40 or more degrees of the thermometer, experienced in the course of a few hours, between mid-day at Salenche, and evening at the foot of the Glaciers in Chamouni. There were upwards of fifty travellers here, many of whom were females and invalids; yet none suffered inconvenience from this rapid atmospheric transition. This was still more remarkable in the journey from Martigny to the Great St. Bernard. On our way up, through the deep vallies, we had the thermometer at 92° of reflected heat for three hours. I never felt it much hotter in the East Indies. At nine o'clock that night, while wandering about the Hospice of the St. Bernard, the thermometer fell to six degrees below the freezing point, and we were half frozen in the cheerless apartments of the monastery. There were upwards of forty travellers there—some of them in very delicate health; and yet not a single cold was caught, nor any diminution of the usual symptoms of a good appetite for breakfast next morning.

"This was like a change from Calcutta to Melville Island in one short day! So much for the ability to bear heat and cold by journeying among the Alps. Let us see how hygrometrical and barometrical changes are borne. A very large concourse of travellers started at daybreak from the village of Chamouni to ascend the Montanvert and Mer de Glace. The morning was beautiful; but, before we got two-thirds up the Montanvert, a tremendous storm of wind and rain came on us, without a quarter of an hour's notice, and we were drenched to the skin in a very few minutes. Some of the party certainly turned tail; and one

Hypochondriac nearly threw me over a precipice, while rushing past me in his precipitate retreat to the village. The majority, however, persevered, and reached the Chalet, dripping wet, with the thermometer below the freezing point. There was no possibility of warming or drying ourselves here; and, therefore, many of us proceeded on to the Mer de Glace, and then wandered on the ice till our clothes were dried by the natural heat of our bodies. The next morning's muster for the passage over the Col de Balme, shewed no damage from the Montanvert expedition. Even the Hypochondriac abovementioned regained his courage over a bottle of Champagne in the evening at the comfortable "Union," and mounted his mule next morning to cross the Col de Balme. This day's journey shewed, in a most striking manner, the acquisition of strength which travelling confers on the invalid. The ascent to the summit of this mountain pass is extremely fatiguing; but the labour is compensated by one of the sublimest views from its highest ridge, which the eye of man ever beheld. The Valley of Chamouni lies behind, with Mont Blanc and surrounding mountains apparently within a stone's throw, the cold of the Glaciers producing a most bracing effect on the whole frame. In front, the Valley of the Rhone, flanked on each side by snow-clad Alps, which, at first sight, are taken for ranges of white clouds, presents one of the most magnificent views in Switzerland, or in the world. The sublime and the beautiful are here protended before the eye, in every direction, and in endless variety, so that the traveller lingers on this elevated mountain pass, lost in amazement at the enchanting scenery by which he is surrounded on every point of the compass. The descent on the Martigny side, was the hardest day's labour I ever endured in my life—yet there were three or four invalids with us, whose lives were scarcely worth a year's purchase when they left England, and who went through this laborious, and somewhat hazardous descent, sliding, tumbling, and rolling over rocks and through mud, without the slightest ultimate injury. When we got to the goat-herds' sheds in the valley below, the heat was tropical, and we all threw ourselves on the ground and slept soundly for two hours—rising refreshed to pursue our journey.

"Now these and many other facts which I could adduce, offer incontestible proof, how much the morbid susceptibility to transitions from heat to cold—from drought to drenchings—is reduced by travelling. The vicissitudes and exertions which I have described would lay up half the effeminate invalids of London, and kill, or almost frighten to death, many of those who cannot expose themselves to a breath of cold or damp air, without coughs or rheumatisms, in this country. These facts may suggest some important indications to the physician who has charge of patients labouring under, or threatened with, certain affections of the chest."

"The next effect of travelling which I shall notice is its influence on the organs of digestion. This is so decided and obvious, that I shall not dwell long on the subject. The appetite is not only increased; but the powers of digestion and assimilation are greatly augmented. A man may eat and drink things while travelling, which would make him quite ill in ordinary life. A strong proof of its effects on assimilation is afforded by the universal remark that, although much more food is taken in while travelling, much less fæcal remains are discharged, and costiveness is a very general symptom among those who make long and repeated journeys, especially in a carriage or on horseback. The motions, which were previously of bad colour and consistence, soon become formed, or even solid, and of a perfectly healthy appearance. The constipation, which often attends passive or mixed exercise, on these occasions, is hardly ever accompanied by any inconvenience; and travellers will go two or three days without a motion, and experience no disagreeable sensation, although the same degree of confinement of the bowels, at other times, would render them ill, or at least very uncomfortable.

"These unequivocally good effects of travelling on the digestive organs account satisfactorily for the various other beneficial influences on the constitution at large. Hence dyspepsia, and the thousand wretched sensations and nervous affections thereon dependent, vanish before persevering exercise in travelling, and new life is imparted to the whole system, mental and corporeal."

"This kind of exercise has a marked influence on the absorbent system, exerting it into great activity. The fluids from the intestinal canal are rapidly taken up into the circulation, and thrown off by the skin—which is one cause of the constipation to which most travellers are subject. This effect of travelling exercise points out the benefits which may be obtained from it in certain tropical complaints, and in obesity. The activity of the absorbents causes the fat to be taken up, while exercise and improved digestion increase the force and firmness of the muscles. Thus corpulent people become thinner while travelling, (especially where active exercise is combined with passive) but what they lose in weight they gain in strength.

"The effects of travelling on the circulation are peculiar. Active exercise quickens, no doubt, the pulse—while passive exercise in a carriage renders it slower. In those diseases of the heart, therefore, where there is enlargement of the organ, with increase of force in the circulation, there can be little doubt, that a combination of active and passive exercise would be hazardous. In such cases the exercise should be entirely passive, and then the effects would be beneficial. There are many cases however, where the irritability of the heart depends on disordered states of other organs, and especially of the stomach—in which cases, the exercise of travelling, active and passive, offers a powerful remedy. In such instances, the exercise should be, at first, passive;—and afterwards the active kind gradually ventured on.

"There is but one other effect of travelling to which I shall allude before I close this Essay, but I think it is a very important one—if not the most important of all. It is the influence which *constant change of air* exerts on the blood itself. Every one knows the benefits which are derived from change of air, in many diseases, when that change is only from one part to another, a few miles separated. Nay, it is proved, beyond all possibility of doubt, that the change from what is considered a good, to what is thought a bad air, is often attended with marked good effects. Hence it is very reasonable to conclude, that the *mere change* of one kind of air for another has an exhilarating or salutary effect on the animal economy. It is true that we have no instruments to ascertain in what consists this difference of one air from another, since the composition of the atmosphere appears to be nearly the same on all points of earth and ocean. But we know, from observation, that there are great differences in air, as far as its effects on the human body are concerned. Hence it would appear that the human body, confined to one particular air, be it ever so pure, languishes at length, and is bettered by a change. This idea is supported by analogy. The stomach, if confined to one species of food, however wholesome, will, in time, languish, and fail to derive that nutriment from it, which it would do, if the species of food were occasionally changed. The ruddy complexion then of travellers, and of those who are constantly moving from place to place, as stage-coachmen, for example, does not, I think, solely depend on the mere action of the open air on the face, but also on the influence which change of air exerts on the blood itself in the lungs. I conceive, then, that what Boerhaave says of exercise, may be safely applied to change of air. 'Eo magis et densum, et *purpureum sanguinem esse*, quò validius homo se exercuerit motu musculorum.'"

As medical men are often consulted as to the propriety or salubrity of a tour on the Continent, the following rapid outline of one, in which some of the most interesting parts of the world were travelled over in the course of three Autumnal months, may not prove uninteresting.

"The experiment was tried, whether a constant change of scene and air, combined with almost uninterrupted exercise, active and passive, during the day—principally in the open air, might not ensure a greater stock of health, than slow journeys and long sojourns on the road. The result will be seen presently. But in order to give the reader some idea of what may be done in a three month's tour of this kind, I shall enumerate the daily journeys, omitting the excursions from and around those places at which we halted for the night, or for a few days. Our longest sojourn was that of a week, and that only thrice—at Paris, Geneva, and Brussels. In a majority of places, we only stopped a night and part of a day, or one or two days, according to local interest. But I may remark that, as far as I was concerned, more exercise was taken during the days of sojourn at each place, than during the days occupied in travelling from one point to another. The consequence was, that a quarter of a year was spent in one uninterrupted system of exercise, change of air, and change of scene, together with the mental excitement and amusement produced by the perpetual presentation of new objects—many of them the most interesting on the face of this globe.

"The following were the regular journeys, and the points of nightly repose: —1, Sittingbourn—2, Dover—3, Calais —4, Boulogne—5, Abbeville—6, Rouen —7, Along the banks of the Seine to Mantes—8, Paris, with various excursions and perambulations—9, Fontainbleau—10, Auxerre—11, Vitteaux—12, Dijon, with excursions—13, Champagnole, in the Jura Mountains—14, Geneva, with various excursions—15, Salenche—16, Chamouni, with various excursions to the Mer de Glace, Jardin, Buet, &c.—17, Across the Col de Balme to Martigny, with excursions up the Valais—18, By the Valley of Entrement, &c. to the Great St. Bernard, with excursions —19, Back to Martigny—20, Vivian, on the Lake of Geneva, with excursions —21, Geneva—22, Lausanne, with excursions—23, La Sarna—24, Neuf-Chatel —25, Berne, with excursions and perambulations—26, Thoun—27, Valley of Lauterbrunen, with various circuits—28, Grindenwalde, with excursions to the Glaciers, &c.—29, Over the Grand Scheidec to Meyrengen, with excursions to waterfalls, &c.—30, By Brienz, Lake of Brienz, Interlaken, and Lake of Thoun, with various excursions, to the Giesbach and other waterfalls, back to Thoun —31, Berne—32, Zoffengen—33, Lucerne, with various excursions—34, Zoug and Zurich—35, Chaufhausen, and Falls of the Rhine—36, Neustad, in the Black Forest—37, By the Vallée d'Enfer to Offenburgh—38, Carlshrue, with excursions —39, Heidelberg—40, Darmstadd—41 Frankfort on the Maine, with excursions —42, Mayence, with excursions—43, Coblenz, Bingen, Bonn, &c.—44, Cologne—45, Aix La Chapelle, with excursions—46, Liege—47, Brussels, with a week's excursions—48, Ghent and Courtray—49, Dunkirk—50, Calais—51, Dover—52, London.

"Thus, there were 52 regular journeys during the tour, and 32 days spent in excursions and perambulations. And as there never was so much exercise or fatigue during the journeys as during the days of sojourn and excursions, it follows that the whole of this tour might be made with great ease, and the utmost advantage to health, in two months. As far as natural scenery is concerned, it would, perhaps, be difficult to select a track, which could offer such a succession of the most beautiful and sublime views, and such a variety of interesting objects, as the line which the above route presents. It would be better, however, to dedicate three months to the tour, if the time and other circumstances permitted, than to make it in two months; though, if only two months could be spared, I would recommend the same line of travel, where health was the object. Perhaps it would be better, however, to reverse the order of the route, and to commence with the Rhine, by which plan the majesty of the scenery would be gradually and progressively increasing, till the traveller reached the summit of the Great St. Bernard, or Mont Blanc.

"The foregoing circuit was made, as far as the writer is concerned, entirely in the open air; that is to say, in an open carriage—in char-à-bancs—on mules—and on foot. The exercise was always a combination, or quick succession of the active and passive kinds, as advantage was always taken of hills and mountains, on the regular journeys, to get down and walk—while a great part of each excursion was pedestrian, with the char-à-banc or mule at hand, when fatigue was experienced. This plan possesses many advantages for the invalid, over the purely active or purely passive modes of travelling. The constant alternation of the two secures the benefits of both, without the inconveniences of either. As the season for travelling in Switzerland is the hottest of the year, and as, in the valleys, the temperature is excessive, so, great danger would be incurred by the invalid's attempting pedestrian exercise in the middle of the day. But by travelling passively in the hot valleys, and walking whenever the temperature is moderate or the ground elevated, he derives all the advantage which exercise of both kinds can possibly confer, without any risk to his health."

In the above tour, the journeys varied from 20 to 50 or 60 miles in the day, and were always concluded by sunset—often much before that period. The usual routine of meals was—some coffee at sunrise, and then exercise, either in perambulations, excursions, or on the first stage of the day's journey. At noon, a *dejeunée à la fourchette*, and then immediately to exercise or travel—concluding the journey, and the exercise of the day, with dinner at the 8 o'clock table d'hôte, where a large company of different nations was usually collected. By ten or or eleven o'clock, all were in bed—and all up again at six in the morning. A tour of this kind is well calculated to restore health, in many diseases, by insuring a more regular system of exercise than can be effected under any other circumstances.

